# Special Meeting of the Fulton County Medical Society for the Discussion of the Social Evil

**Published:** 1907-12

**Authors:** 


### SPECIAL MEETING OF THE FULTON COUNTY MEDICAL SOCIETY FOR THE DISCUSSION OF THE SOCIAL EVIL.

Report of Committee on Sanitary and Moral Prophylaxis.

---

In compliance with the request of the Fulton County Medical Society, the Committee appointed sometime ago has arranged for a discussion of an important although somewhat unsavory question, but the good that may be derived from a judicial consideration of this subject justifies, we feel sure, the special meeting tonight. Excellent work is being done along this line in the large cities of the United States and Europe and we think that Atlanta should do her share in mitigating at least some of the evils, disease and suffering that come from ignorance and carelessness concerning these matters.

We have invited the lawyers, ministers, physicians and students of the City, as well as some of the men prominent in business affairs, to be present with us tonight and some of them have agreed to make short addresses upon a general consideration of this subject. At subsequent meetings we hope to take up special features.

Although most of the physicians present are familiar with the facts to which we wish to call attention, we thought it expedient to quote a few of the statistics bearing on certain points in order to give the other gentlemen an idea of the scope and magnitude of this so-called social evil, in whose wake we find gonorrhea and syphilis with their widespread and disasterous results urgently demanding our attention.

Neisser says that, with perhaps the exception of measles, gonorrhea is the most common of all diseases. Morrow asserts that it is even a more potent factor of depopulation than syphilis as a large per cent of involuntary childlessness is due to it. He also thinks that the extent to which it prevails in private life is much greater than is generally supposed and he makes the startling statement that there is more gonorrheal infection among virtuous wives than there is in professional prostitutes. The mild chronic conditions, or those that apparently have been cured, but in whom infection of the deeper structures as the prostate gland or seminal vesicles still persists, accounts for the larger part of marital gonorrhea.

Authorities who have studied this subject vary widely in their estimate of the per cent of those who have or have had gonorrhea; the number being 50 to 90 per cent of the adult male population of the world. Even the lowest estimate given by the ultra-conservative is still sufficiently large to justify or demand our serious attention. Schwartz has calculated that ten per cent of married men in large cities enter wedlock with a chronic yet infectious gonorrhea.

The monetary loss is so great from gonorrhea alone and the statistics so meagre and unreliable that I will ask you to make a mental calculation and estimate about the average cost of an average patient for necessary expenses incident to the disease, and to that add the loss of time, the loss in physical weakness and mental worry and the loss to the Nation from lessened capacity for production. The census of 1900 showed that there were about 20,000,000 men in the United States between 20 and 55 years of age. The average estimate of those who have or have had gonorrhea is between 70 and 80 per cent of men. This would give us between 14,000,000 and 16,000,000 who have contracted this disease. Now multiply this by the average loss of each patient to himself and the state and you will have part of the monetary loss from gonorrhea.

It is under the head of operations and invalidism in women, however, that we see the appalling results of gonorrhea. Price and Humiston claim that from 90 to 95 per cent of the abdominal operations performed in the world today on women are required on account of affections, adhesions and pus collections due to gonorrhea. These figures are higher than those given by other authorities, but even the lowest is enough to stir the most indolent to activity when this amount of innocent suffering is contemplated. Equally as frequent as the operations are the patients who are made invalids for life, and worse than these are the women who become mentally unbalanced on account of the removal of their sexual organs which were so damaged that the gynecologist found them useless or a menace to their lives. Many of these are due to latent gonococci in apparently cured husbands.

Through its effect on the epididymes and prostate gland, gonorrhea plays the main role in producing sterility and impotence in men. Benzler followed the history of 474 soldiers with gonorrhea and found sterility resulted in 10 per cent of the cases of urethral involvement, in 23 per cent where there were unilateral epididymitis and in 41 per cent where the epididymitis was double.

Neisser says that 50 per cent of involuntary childless marriages are due to gonorrhea. The catarrhal condition of the mouth of the womb prevents the entrance of the spermatozoa thereby rendering the women sterile. After the inflamation has reached the l ining of the uterus and the tubes, still further obstruction prevents the fertilization of the ovum,even when the disease has not progressed to pus formation. Bland-Sutton says that gonorrhea is the chief cause of sterility in the prostitute class. Some of the worst results of gonorrhea are due to the secondary infections made possible and favored by the inflammation of the mucous membranes.

Formerly about 50 per cent of all the blindness was due to gonorrhea but since the adopton of Crede's prophylactic measures this has been reduced to about 20 per cent. Even with this reduction it may still be given as evidence against gonorrhea. It is claimed by reliable authorities that 80 per cent of ophthalmia in infants is due to gonorrhea.

### SYPHILIS.

Syphilis is more greatly feared but less frequently encountered than is gonorrhea. From 10 to 20 per cent is considered a fair estimate of those who contract this disease. A recent issue of the "Canada Lancet" stated that in the British Army syphilis costs the Nation about $15,000,000 annually for sickness and about $20,000,000 in pensions and superannuations, or a total of $35,000,000 a year.

Investigations in the hospitals for insanity in France have shown that from 25 per cent to 39 per cent of the deaths in these institutions may be traced to syphilis. About 90 per cent of locomotor ataxia is caused by syphilis. In fact the chief danger from it comes not immediately, but later in life from its involvement of internal organs essential to life as the brain, spinal cord, heart, arterial system and liver.

Syphilis is frequently introduced into married life and is exceedingly likely to cause abortions. Morrow says that 60 to 80 per cent of syphilitic children die before being born or very soon after birth. Keyes claims that gonorrhea destroys the race, either by sterilizing a potential parent or by blinding a new born infant; while syphilis leaves the parent neither sterile nor impotent; the child it may kill or leave with indellible disfigurement, or it may leave it liable to destruction by other agencies, notably tuberculosis.

He also states that the Actuarial Society of America estimates that the acquisition of syphilis by the average American, decreases his expectancy of life about one third. The Surgeon General's report for 1905 shows that in the United States army syphilis stands ahead of every other malady as a destroyer of careers.

It is estimated by men familiar with the conditions, that there are 150,000 syphilitics in Berlin and 200,000 in New York.

A large number of views and statistics could be quoted to bring out many other phases of the subject, but we are to hear from several others and we think that the facts just given show sufficiently clearly that our "campaign of inactivity" should be abandoned and that something should be done to mitigate at least, this evil.

The Committee therefore suggests that other meetings be held at stated times and that an effort be made to obtain the cooperation of individuals and organizations working along this line.

E. G. Ballenger,
Theo. Toepel,
Archibald Smith.

---

### POT-POURRI.

BY DR. E. C. CARTLEDGE, ATLANTA, GA.

To the much reading and experience of my audience, anything I could say might savor more of a mixture or mess than rose leaves; but I shall tell you of things or experiences good to me and to some of you, I trust.

1st—I have found a saturated solution of Aluminum Acetate of very great value as a penetrating astringent and anti-inflamation application to areas of redness, heat, pain and swelling. It may not be so good as Ichthyol in erysipelas. It is too astringent to use as we do a 25 per cent Listerine continuous moist dressing in crushed masses of bone and tissue of exterernities to bring about healthy demarcation between viable and destroyed tissues. but where we have the signs of inflamation as above given it excels when used in saturated solution in continuous moist dressing until tissues pucker and bleach and reduce until you recognize sufficient effect. Dr. Oscar Lyndon was the benefactor to my patients in the above. Old fashioned clay and vinegar poultice must furnish us the same thing.

2nd—X-Ray has relieved an exophthalmic goitre for me. I have always failed utterly on several before. My Aluminum Acetate failed to penetrate and astringe the Goitre. I used the X-Ray with the idea of contracting the Thyroid tissues, for I had noticed this effect of the ray, especially in a case of breast cancer sent to me for X-Ray treatment after a Halstead's Operation by Dr. Kime, in which the contractile scar and tissue was felt to draw the more as I used the ray. Also its effect to absorb effusions as in Pleurisy and its effect on lymphoid tissue, suggested its use. The only symptom this patient had recognized, and for which she left the Quack advertisers and came to me, was excessive, constant sweating, even in cold winter.

She wore some water-proof lining under her outer garments at places to save her embarrassment. This symptom was relieved after eleven treatments. Her heart beat was 90. Is now 78. She suffered no exopthalmos. Thyroid was enlarged so little she had not noticed it, tho' it was easily apparent. It has reduced very much. Going in swimming a few weeks ago brought on another attack of sweating four months after treatment. She kept up her swimming more moderately and had no more sweating. She said the thyroid was much enlarged and pulsating after the first effort in learning to swim, but soon subsided and she remains well of sweating. I have not seen a more happy result; she bad suffered from this profuse sweating from 12 years of age until now 26, and of which she complained most in winter. She is completely free from it. The patient dated her sweating from getting over-heated at running and playing at school, tho' heredity plays its part. Her father had Ashhma and her mother will not ride on street cars or elevators. The patient weighs 160 pounds and is a picture of robust health.

(This case suggests another case of sweating which I have mentioned before I think. A young man sweated very freely all the time, so he said, and he looked it, and had lost thirty pounds in weight. A druggist fitted him with a suspensory and relieved him after I had tailed to find the cause of his sweating.)

I have cured every epithelioma I have treated. I recall eight cases tonight. I have failed on every lupus, three in number. I think it a mistake and a contradiction in treatment to cover these little or large face cancers with adhesive plaster or other dressing that shuts off the day and the sunlight. I've had one patient shave off mustache which showed an epithelioma on the upper lip. I cured one such cancer with suns rays concentrated by a magnifying glass. Some physician in California reported doing the same. The X-Ray tans like sun's rays and blisters like when you were a boy and went in swimming and your mother put cream on your blistered back. Therefore, I would not lose the aid I feel sunlight to be in cases treated by -Ray.

3rd—Schleischs Infiltration Anaesthesia is the best local anaesthetic, I think. I notice many physicians still use some per cent of cocaine.

The formula is Cocaine Muriate grains iii, Sodium Chloride, grains iii, Morphia Muriate, grains ss, water, ounces iii ss. It maintains an anaesthesia longer than 2 per cent cocaine. It is about one fifth of 1 p.c. Cocaine and so can be used freely even about the mouth and head without danger of toxic effect of cocaine. It doesn't give the inflammatory reaction that cocaine gives. This formula has been in our medical works for years but I still frequently read, in Medical Journals especially, about using two or four per cent cocaine. Under this anaesthesia I've worked 45 minutes on scrotal amputations with resection, and operated extensive fistulas in ano, removed neck glands, etc.

4th—I see an occasional non-union in fractures where wooden or other pressure splints have been used, and in which cases I think plaster paris splint would have served better. In nearly all fractures, of extremities, especially bone shafts, just an accurate an opposition can be gotten with plaster paris, and better maintained, and besides comfort, it allows a quite free blood circulation for the needed repair of the bone. "The life of the flesh is the blood thereof." A splint of board, rubber adhesive plaster and bandage create a pressure that greatly interferes with the circulation and ready repair.

The mobilizing method hasn't inspired me with confidence to try it.

5th—Carbolic Acid can be better neutralized with spirits of Camphor or Campho-phenol locally than with alcohol. Camphor combines with it very much more quickly. The only case of Carbolic Acid swallowing I've treated, I gave equal parts of spirits of Camphor and water. You have in this as strong an alcoholic as can be swallowed, equalling whiskey, and the greater value of Camphor. I followed this with Camphorated Oil.

6th—Absolute supine rest to a heart losing its hold on circulation is vastly superior to any drug and all of them. The Insurance examiners note that a heart beating 72 sitting, goes to 80 standing, putting 9 more work on it. Yet I have seen a man walking up and down seven steps and length of two rooms ad libitum two days before he died, with his heart struggling wildly dilated, leaking and taking heart stimulants to relieve him.

In giving Iodide of Potash, I learn of many people who have taken it poorly and with much upsetting of the stomach by taking it after meals or with milk.

I have very many times been confirmed in the wisdom of giving such medicines on an empty stomach an hour or two before meals with a good amount of water. Give the dose in a glassful of water, followed by a glass every fifteen minutes until three to five full glasses are taken.

The water is a most valuable part of the treatment, whether in syphilis, asthma or rheumatism. This method is thrice good. (1) It supplies at the right time the water needed—itself an alterative and eliminant and serving more surely to convey the medicine into the blood and tissues. This dilution saves the stomach irritation better than a meal will do. (3) It does not disturb an always important digestion as when mixed with food.

Another cause of disturbing a stomach with Iodide Solutions, is that such solutions when exposed to light for long, precipitate Iodine which is very irritating and is remedied by clearing up your solution by adding Dil. Hypophosphorus Acid to redissolve your Iodine (as demonstrated to me by Mr. R.C. Hood of Southern College of Pharmacy) or by always getting a fresh solution. Some Pharmacists keep a stock solution ready made up. This is why patients tell us that there is a difference in getting their Iodide prescriptions filled at different towns or stores.

In this way no hindrance is experienced in taking your patient up to 90 to 175 or more grains three times a day and getting some of the happiest results of medicine giving.

### DR. THEODORE TOEPEL.

The question of "Moral Prophylaxis" is a vital one to every community which has the welfare of humanity at heart and should receive far more attention than has thus far been bestowed upon it. This reform movement must be carefully planned and all phases of its future results must be well considered before presented to the public at large. All great reforms progress slowly, especially when they touch social conditions which are deeply rooted and hard to eradicate. It will require much patience and great perseverence on the part of those who push this campaign. But success is inevitable, if we can now lay the foundation, planning to inform the people as to true conditions of the so-called "social evils," gonorrhea and syphilis. Let the next generation reap the benefits of this, our work; it will be all that we can accomplish. Do not let us delay any longer. Let us get to work to enlighten the people in the necessary prophylaxis. Do way with false modesty, which in my opinion is the beginning of the existing precarious social conditions, and call a spade a spade. I believe it is right that the instruction given the people on this subject must emanate from the medical profession. Mass meetings should be called for men and for women. Here men and women of high moral character should address the audience, holding up before their hearers the dangers of these diseases in such a manner that no doubt would remain as to the sincerityof the speakers in desiring physical and mental uplifting. Fathers should learn enough of this subject so as to be able to talk to their sons in a plain, upright manner about the sexual intimacy existing between man and woman, warning them of the dangers awaiting them in illegitimate coition. Mothers should learn that it is right to tell their daughters of the physiological functions of the sexual organs and their hygiene. Let it be urged that they speak to their daughters of the high duties of motherhood, of the care of themselves and of the expected offspring, especially when their daughters are about to enter matrimony themselves.

We should distribute literature on the evils of sexual diseases among the people regardless of social differences. This literature should contain in plain unvarnished words what is necessary to know to check the spread of these diseases. Parents must first be educated before they can educate their children. Their children should receive the first lessons in moral prophylaxis at home by example in right living and teaching. Teach children of the grammer schools all the hygiene that pertains to the well being of the body and enough physiology so that they know the uses of every vital organ and its care. The subject of cleanliness should be of paramount importance in every talk on hygiene. In the Boy's and Girl's High Schools, the uses of the vital, as well as the sexual organs, should be discussed in an unbiased manner from a hygienic and physiological standpoint. Information regarding diseases of the sexual organs could at this age of the students, be well introduced and intelligently presented without creating the slightest misconstruction by the students.

Cell-life ought to receive due consideration at this period of instruction in physiology.

At colleges, regular lectures should be given to the young men and women covering fully all the dangers of these and allied diseases and their prevention.

I realize that this plan of campaign is a big undertaking, but in my opinion it is the only proper course to pursue in order to reach the young and the old, and there-by to slowly but surely improve the physical and moral condition of mankind.

### DR. R. R. DALY.

Gentlemen:—

In the prevention of promiscuous and excessive coition and the diseases resulting therefrom, there are lines to follow and obstacles to overcome that have not been noted tonight.

Father Gunn has very justly called attention to the bodily condition of the children so far as cleanliness and proper hygienic surroundings are concerned and indicated the need of these to make a boy clean thinking. He says truly that the thought precedes the act.

It is pertinent to note how a boy thinks and what may hinder him from the clean thoughts of normal healthy life. Without going into psychology to a burdensome extent, let us note that the boy thinks about what he sees, hears, feels and generally senses and that the material of his thoughts is necessarily made up of what he perceives. It is obvious that when anything is wrong with his perceptive organs, his thoughts are limited or hampered. The mind, shut off from seeing or hearing, or one in which perceptions of sight and hearing are distorted by reason of improper working of these sense organs, must use what material it has and is turned in upon itself for meditation. And here is a point I wish to emphasize. The healthy normal boy by sensing everything as he should, does not have time for meditation and introspection. He is alert, busy and happy when awake and tranquil in his sleep. The boy who doesn't see well, can't follow the sports of his fellows. He becomes in a measure unsocial and turns back upon himself for his amusement. If he has heard putrid tales, if he has been taught onanism, if his sexual organs have been excited by a bad nurse, if he has local irritation due to phimosis, rectal trouble or untidiness, his thoughts go to the primal factors and the procreative instinct dominates them. Such a boy grows to gratify his desires and to be controlled by them. To have a boy think well he must have properly acting perceptive organs. He must breathe well and have his blood cleansed and enriched at every breath as much as possible. He must sleep well and be free from all peripheral irritations.

The value securing these conditions has upon conduct has been demonstrated in the truant schools of New York, Chicago and Pittsburg where many incorrigible boys have been delivered from self bondage by opening up their environment to them. Defective vision has been remedied with glasses; adenoids removed from the naso-pharynges and diverted septi straightened. Parasthesias have been recognized and relieved and circumscisions performed with strikingly beneficial results. Give the boy his normal condition, establish his normal relations to a decent environment and I care not so much what heredity may be, he has more than a fighting chance to grow up clean minded and self controlled.

I have spoken of the boy because he has the usual aggressive element, but the same thing should be said of girls for similar reasons. Those of us who have had profesional relations with girls' schools know that erotism invades them and damages many a child.

The imperfections of our creation are numerous and more and more striking as science extends our vision. It is our duty to remedy them wherever possible if we want the generations to improve.

The psychic factor just dwelt upon blends closely with the Sociologic one of our civilization and its conventions. Our ethical evolution seems at times to diverge from the physical, but of course the swerving cannot be great without destruction. Men were evolved through a stage of free love. It makes no difference when it was or what the details were. Our civilization decrees that a man shall have but one wife at a time. It inculcates that he shall not have intercourse with women unless he is married It places restrictions upon the young men at the lustiest period of their lives and says "Thou shalt not."

Far be it from me to criticize the wisdom of these conventions, but it is within anyone's province to note what happens.

Conventions are of time. The procreative instinct and sexual desire are of eternity so far as mans history is concerned. From the first awakened desire there is a conflict between it and the conventions till death parts them among many men. The conventions are there written as large as you please. The desire is here present, potent, practical. It doesn't have to be read, it interprets itself and dominates many times.

No matter what teachings there may have been, no matter what hygienic efforts have been extended, the great primordial desire bursts through them all and wins. Wins! Yes, because wherever there is the "Masterfu' Man," as Barrie calls him, there shyly waiting is the yielding woman.

Not all men are so dominated, but we must recognize as always present a considerable group of them and they way seem to interfere with the work. It is well to look forward and prepare for all recognizable difficulties before entering upon a struggle. Once seen in advance they are not so discouraging close at hand. Even this difficulty may resolve itself when we think that what we are after is the prevention of disease fundamentally and that once that is eradicated, morals will have a better chance.

One more matter that has not been spoken of. The stress of living and the increased demand of social life deter young men from early marriage on the one hand and make them childless on the other. Because a man may not marry is no reason why he should remain virtuous. He knows the expense of domiciled life and can not meet it. He doesn't always know the expense of bowing to Priapus, but he takes his chance. The man who does marry, soon enough faces the question of the prevention of conception, and his troubles thicken about him. Maybe all goes well enough for a long time and he doesn't understand why his wife is not as strong and well as she was before marriage. No operation has been performed ,nothing definite is present, but there is a difference. The home is not what he wanted it to be—not what she wanted it to be. Again injuries are more apparent and the man knows that the relations as he has tried to have them, are dangerous; the very fear drives him from his bed. All these conditions lead to illicit intercourse, give their support to prostitution and furnish avenues to the dissemination of venereal disease.

What can be done about it? First, through the public schools the defective children can be reached. Municipal regulation can effect that; then the parents will be interested. These can be taught by circular letters issued by some local society as is now done in Chicago. A similar method may be used when the time is ripe to inform young adults of their responsibilities and dangers. Let the matter be information, and not exhortation. The leading professions can teach these facts more frankly and freely than they have before and be more ready to offer counsel. Thus gradually a public sentiment in this matter may be built up and then leaders can strengthen that sentiment by spreading further information. Public meetings may be held, and other classes—the ignorant and depraved—reached. The next generation should show improvement and that is all we can do. We can hope the work will go on, not to Utopian conditions, but to reasonable ones in accord with knowledge, common sense and the better state toward which civilization is turning out its best.

### EX-GOV. W. J. NORTHEN.

Mr. Chairman:—

It was not at all my expectation to be called upon to discuss the matters now up for consideration by this intelligent and well informed body of Physicians. You have taken me quite unexpectedly now that you invite me to speak at the close of your discussion.

Except as an interested citizen, I could not feel at all inclined to express my views on so important a matter as the Social Evil, in the presence of capable professional men who are far better informed upon its origin and influence than I can ever hope to be.

You are supposed to know, and I certainly give you full credit for knowing thoroughly well, the grave and serious matters you have been discussing for the past hour or more. During this time you have been opening up fundamental social questions in a way that has filled me with wonder and alarm. It never entered my mind to conceive, as you have presented it, the general prevalence and the dread dangers of the so-called social sin. If these things are undermining our social fabric to the alarming extent repeatedly stated in this presence this evening, I deliberately charge that you will be criminal before God and the people of our City if you do not have all of us know to the fullest extent, that you know, the dangers that confront us, because of this social sin. If what you have said is true, and I do not doubt one word of your utterances, there ought to be great and glaring red lights before the eyes of every man in Atlanta, including all classes, so that all of us may know and apply the remedies, before destructive evils thoroughly poison our whole social system.

Why not have your audience enlarged and fill the capacity of the Grand Opera House with men that you may tell again to them the evils and the remedies for the conditions and the dangers that are threatening the community?

If the so-called Social Evil is so destructive of the young, why not make proper instruction upon this subject a part of our school system, and have the younger generation of both sexes, fully informed as to the injurious effects that follow impurity and moral uncleanness that seem to be so common over the land? With these evils so alarmingly prevalent and so destructive of mind, body and spirit, we can but be cruelly unkind if we do not thoroughly inform the youth of the City, before they shall be ignorantly destroyed.

When you say that thirty to forty per cent of the blindness among the unfortunates of this class of our people is to be attributed to the destructive force of the so-called social evil, how can we delay to inform all men, lest we allow, purposely allow, the ruin of the race and approve the open shame of the home?

When you tell me that fifty to seventy-five per cent of the male population of the larger cities is more or less afflicted with this moral leprosy, you challenge my faith in the people if I retain my confidence in your statement and my regard for your opportunities to know.

All these things being true, as your discussion must lead us to believe, I charge you, in the name of clean living, and in the name of a humanity that seems going steadily and rapidly to decay, lay aside all false modesty, call these dreadful things by their horrid names, and, with heroic effort, begin the recovery of the generation through the purity of individual life and the sanctity and virtue of the homes of all the people.

By all means, do not let the matter remain as it now is, but call another and a larger meeting and tell again all you have said tonight, to all the people, who can be induced to come. We should speedily redeem the City from the curse of this sin.

### DR. R. R. KIME.

Was glad to see this meeting and hear this discussion as it is what he had desired for sometime. It is one of the most important social questions of the day, almost as serious as tuberculosis in ultimate results. The public, and especially the youths, should be educated on this subject and its dangers. The false notion of two standards, one for men and one for women, is doing much harm and should be abandoned. The idea that continence in men is injurious to health should be condemned by all right thinking people. Men can and should live continent lives, as it conduces to manly development in youth and later conserves their health and strength and prevents disease and degeneration that results from immoral and illegal indulgence. The normal function of these organs in youth is to properly develop the body, later to procreate the race and any immoral, illicit or excessive use destroys mans usefulness and influence to that extent, beside lowering the vitality of the individual and transmits disease and degeneration to innocent persons and their offspring.

The instruction of men as to the results of gonorrhea in the male, that the majority of cases are never cured, that the majority of operations for suppurative disease of pelvic organs in women is due to infection from the male, and that 15 to 20 per cent of the blind children are due to uncured gonorrhea in the father; that he himself may suffer with rheumatism, disease of the heart, suppurative disease of the bladder, kidneys, etc., as a result of gonorrhea, would deter many from taking such risks that cause so much disease and suffering. What man knowingly desires to produce blindness of his own child, disease or death of his own wife? Education is the remedy; but how shall we educate the boys and girls? The majority of parents do not know how and many, especially the men, have not so lived themselves that their teaching would have weight or moral influence. Boys and girls should be taught the hygiene and physiology of sexual organs in a clean, physiological, moral way, later the pathology and diseases that follow violations of nature's laws.

If this can be properly done in the home, so much the better; if not, it should be done in the high schools and colleges, boys and girls being taught separately.

If lectures are given by physicians, they should be selected on account of their high moral standing and fitness, so that their teaching will be not only by precept, but by example in which the students will have confidence. A pamphlet, properly prepared, for boys and girls separately, and placed in the hands of a proper instructor in the High Schools and colleges, and in the hands of parents for home instruction would help materially to settle this vexed question.

### NOW FOR A MEDICAL LIBRARY.

This present age, more than ever has been the case in the past, is one of *Competency*. No matter how high the stand taken by a physician at his graduation, no matter what his ability or advantages, unless he keeps in touch with the work done by the leading investigators throughout the world, he is sure to lag behind his more aggressive competitors who abhor a rut and are willing to advance or make changes in their pet ideas or favorite treatment when an improvement has been scientifically and conservatively advanced. There is but one way to keep up with the profession and that is through medical journals; books, as a rule, are much behind the times, nor can they ever catch up.

Nothing can add so much to ones interest in the diagnosis and treatment of disease as a thorough familiarity with recent observations. This cannot be obtained from a few medical journals even though they are voluminous and first class. No one can be expected to read everything, but everyone should have constantly before him some subject in which he is especially interested; his interest can always be increased by reviewing the recent literature upon that subject and his knowledge augmented thereby.

It is to place the best medical literature within the reach of Atlanta and Georgia physicians that a movement has been projected to build a medical library in Atlanta and arrangements have been made with the Trustees of the Carnegie Library to furnish a suitable building with reading rooms, stocks and an assembly hall if the physicians will buy the lot in the rear of the library and agree to subscribe $500 annually to purchase books, periodicals and binding files for the journals. The City further agrees to furnish heat, light, janitor service and a trained assistant to care for the technical side of the work.

The Committee appointed by the Fulton County Medical Society has secured the option on the lot in question for $7,250.00. By such an arrangement, the medical society will have permanent quarters in which to meet. This medical building will afford a common meeting place for physicians in a central portion of the City and will undoubtedly foster good feeling and friendship among the members of the profession.

Much credit is due Dr. Michael Hoke for his untiring efforts to accomplish what is now within our reach, and it will seem astounding if there is not sufficient "esprit de corps" among the doctors of Atlanta to carry this matter to a successful termination. It is desired that a cash payment of $5.00 be made and the balance be made in four notes, payable six, twelve, eighteen and twenty-four months.

Every physician in Atlanta should contribute to this worthy cause and then feel a personal interest in the library and club rooms to promote his professional ability and standing.

---

### SURGICAL TREATMENT OF DETACHED RETINA.

BY. G. W. MASER, PARSONS, KANSAS.

Four years ago I reported at the Wichita meeting of the Kansas State Medical Society, a case that had been operated upon for detachment of the retina, with the result of restoring sight. This case has remained cured, the sight continuing good to the present time, and must be regarded as a permanent cure.

This year I have done the operation six times on two cases. These cases were of more than ordinary interest on account of the number of operations made on two eyes before reattachment was secured. The conclusions reached were that we should not be discouraged by a failure from a single operation, but should make a number of them, should failure or partial success be the result of the first. Again, the site of the operation is so far removed from the important eye centers, that is, the ciliary region, that the danger is reduced to the minimum. With ordinary care the danger is not nearly so great as would attend an operation for cataract, or even an iridectomy.

The results of the treatment of detachment of the retina have been most discouraging, the cures, if any, have been spontaneous and not the result of any form of treatment. A careful research of the literature on this subject fails to describe the operation as made on these cases, the report of which follows in this article.

I do not claim priority for this operation. It has been copied from the operation made by Mr. Lang of the Royal Ophthalmic Hospital, England. The results have been so encouraging that the method should become more widely known to the ophthalmologists of this country.

Mr. Lang had operated on two cases, the operations being in the nature of an experiment, while at the same time not subjecting the patient to any risk as they were practically blind, and no other plan of treatment offered any chance of success.

The result of the operation on the above two cases I do not know, on account of the short time I had of observing them.

The class of cases operated upon must be selected. No good results would be expected from a detachment caused from a tumor, be it benign or malignant. The operation could only be successful where the detachment was caused by the presence of serum between the retina and choroid, and before the retina had lost its function. Just how long the retina will retain its function after detachment, is not known, but in one of my cases the operation was made four years after the diagnosis of detached retina, with considerable improvement in sight. This leads us to recommend operative treatment of detachments of long standing, if there is fair light perception.

The causes of detachments are, as a rule, obscure. Aside from those caused from traumatism and tumors of different kinds, as a general rule no cause can be found, and whatever theory is advanced remains only a theory, and the pathologist has still to find the real cause. When this is found, we may determine the proper medicinal treatment. We do know that it is more common in males than females. It is more frequent in myopic eyes, owing to the stretching of the choroid and schlera. Some authors state that more than fifty per cent of the cases occur in myopes. It may be found in cases of general debility, when the entire system is weakened from whatever cause.

Various methods of treatment, both medical and surgical, have been tried, but the results have been disappointing. Most cases have been treated by the use of diaphoretics, chiefly pilocarpine, administered hypodermically, together with bandaging the eyes, and the recumbent posture Setons, blisters and leaches have been used with little or no success. As far back as 1859 Sichel introduced the plan of tapping the subretinal fluid by a puncture of the sclerotic and choroid.

In 1863, Von Graefe recommended discession of the retina, in three years had employed it fifty times, but had no lasting benefit in a single case.

De Wecher, in 1877, tried to keep up a continuous drainage of the subretinal fluid by passing a fine gold wire through the sack and leaving it in position, thinking the retina would become reattached, but in place of good results, he found that this treatment sometimes caused sympathetic disease in the other eye.

In 1890, Galezonski recommended the plan of entering a catgut beneath the retina and stitching the retina to the choroid. This plan was a failure. The same author tried aspiration with a syringe, but the plan was a failure. He also tried an iridectomy without success.

Deutchmann, in 1895, proposed the operation of cutting with a double edged knife, the sclera, choroid and retina, into the vitreous space, pushing it to the opposite side of the eyeball, cutting to both sides. His aim was to divide any adhesions or bonds that might be formed in the vitreous body. No statistics are given of the result of this treatment.

Fox, in his work on the eye, reports no cures by any method of treatment.

Norris and Oliver report 789 cases, with no cures by surgical treatment, and only two cases regaining function by medicinal treatment.

Noyes, Juler and De Schweinitz report no cases cured from operation, neither does Mittendorf.

Roosa reports improvement in some cases, but recommends operative treatment only after other treatments have failed.

Nettleship does not report cases cured by operation, but recommends it in recent cases.

Ball reports cases cured by Staerkle of Basel by means of injections of salt solution, the strength being from 2 to 10 per cent. He treated twenty-three cases, having improvement in twenty cases and complete reattachment in three.

Dor of Lyons, claims fourteen complete recoveries in twenty-one cases, using a 20 per cent salt solution. The author does not state whether the injections were made in the vitreous or not.

The operations that I have made were for the purpose of creating a ragged wound in the retina to produce inflammation, with exudation enough to unite the retina to the choroid. Following the operation, the eyes are bandaged and the patient is to lie quietly in the recumbent position for about one week.

The different steps in the operation are, a triangular flap is made in the conjunctiva at a point between the attachment of the recti muscles. After the hemorrhage has ceased, and the clear sclera is seen, a Von Graefe cataract knife is pushed through the sclera, choroid and retina, well into the posterior chamber, then, by turning the knife slightly, the serum is allowed to escape. When the serum is drained, by using the point of puncture as the fulcrum, the retina is further incised. Turning the knife as near to the right angle as possible, the retina is again incised. In this way, considerable inflammation follows. This is exactly what is desired, and has been followed in all my cases by a reattachment at the point operated upon, but not of the entire retina. That part at some distance from the operation has failed to unite when the separation has involved more than half the retina. A second operation is made after the eye has become quiet and the tension normal. In some cases a third operation has been made before complete union has been established. In no case has there been any dangerous inflammation.

It would seem from the foregoing operations, that the opthalmic surgeon would be justified in reoperating until the reattachment was complete.

The first case I operated on was reported five years ago. This man was 25 years old, and had recently recovered from an attack of mountain fever, was convalescing slowly, when, without any symptoms referable to the eye, found he was blind in the right eye, vision being reduced to 1-200. Ophthalmoscopic examination showed a detachment at the lower third of the retina. The operation was made two weeks after the detachment occurred. His vision three months later was 20-30 and has remained good since that time.

Another patient, Mr. H., age 42 years, occupation coal dealer, had been blind in the right eye for about six years. Had a divergent squint in this eye, pupil normal. Had some kind of an operation made on this eye in Omaha. What was done the patient does not know. The ophthalmoscope showed a detachment of the lower half of the retina. Could see a light when held directly in front of the pupil, but not when held above the pupil.

About six weeks previous to his consulting me, the patient began to see black spots in front of the left eye. Sight in this eye remained good until several days before he came to see me. Could see as well as usual one day, and the next day, was unable to recognize his friends or attend to his business. Ophthalmoscopic examination showed a detachment of two thirds of the retina mostly on the temporal side. Patient was unable to see the flame of a candle when held on the nasal side of the eye, and could count fingers at two feet. Tension normal.

January 30, 1907, the first operation was made on this eye, and one month later he could count fingers at fifteen feet. The retina was attached at the point operated on, but some detachment remained at a lower level. Another operation was made February 16th. This was followed by an increase of vision to twenty-200. On May the 11th. there still remained a small detachment at the lower part of the retina, and the third operation was made. On July 8th., the examination showed a perfect reattachment of the entire retina, the vision being 20-100. At this time an operation was made on the right eye, followed by improvement in vision from perception of light only, to the counting of fingers at six feet. September 27th., examination showed retinal bloodvessels small, blood supply deficient and one third of the retina detached at the lower part. A second operation was made on this date, and on October 1st. the sight had doubled.

The third case, Mr. F. age 53 years, occupation real estate agent, suddenly noticed that he could not see with the right eye. Thinks the trouble came on in the night. Had a detachment of the upper third of the retina. There was no exciting cause found in the history. The man was in good general health and had fair light perception at the lower and inner portion of the eye. First operation was made on May 15th, 1907. On the 23rd. the vision was 20-100. Some detachment remained on the 28th. when a second operation was performed. I did not see the case again until July 3rd. when the third operation was made on a small patch of detachment at the lower part. Examination with the ophthalmoscope on September 17th. showed the blood vessels of the retina atrophied. The retina was attached, with a small opaque patch at the lower part, the optic disc normal, vitreous clear, but the vision was reduced to fair light perception.

I am unable to form an opinion as to the cause of the poor vision. My experience has been most encouraging, having succeeded in restoring sight in three out of four cases operated on. The operation as made, promises good results, especially if made early, while the retina retains its function.

